# Downregulation of the *Escherichia coli guaB* promoter by FIS

**DOI:** 10.1099/mic.0.2008/016774-0

**Published:** 2008-06

**Authors:** Seyyed I. Husnain, Mark S. Thomas

**Affiliations:** F Floor, School of Medicine and Biomedical Sciences, University of Sheffield, Beech Hill Road, Sheffield S10 2RX, UK

## Abstract

The *Escherichia coli guaB* promoter (P*_guaB_*) regulates transcription of two genes, *guaB* and *guaA*, that are required for the synthesis of guanosine 5′-monophosphate (GMP), a precursor for the synthesis of guanine nucleoside triphosphates. Transcription from P*_guaB_* increases as a function of increasing cellular growth rate, and this is referred to as growth rate-dependent control (GRDC). Here we investigated the role of the factor for inversion stimulation (FIS) in the regulation of this promoter. The results showed that there are three binding sites for FIS centred near positions −11, +8 and +29 relative to the *guaB* transcription start site. Binding of FIS to these sites results in repression of P*_guaB_ in vitro* but not *in vivo*. Deletion of the *fis* gene results in increased P*_guaB_* activity *in vivo*, but GRDC of P*_guaB_* is maintained.

## INTRODUCTION

The *Escherichia coli guaB* promoter (P*_guaB_*) regulates transcription of two genes, *guaB* and *guaA*, which together constitute the *guaBA* operon. The *guaB* and *guaA* genes encode inosine 5′-monophosphate (IMP) dehydrogenase and guanosine 5′-monophosphate (GMP) synthetase, respectively, and are required for the biosynthesis of GMP from the common purine nucleotide precursor, IMP (Mehra & Drabble, 1981; Tiedeman & Smith, 1984). P*_guaB_* is regulated by the cAMP receptor protein (CRP) and a putative CRP binding site is centred near position −117.5 relative to the *guaB* transcription start site (Hutchings & Drabble, 2000). Furthermore, transcription from P*_guaB_* is strongly enhanced by an UP element (Husnain & Thomas, 2008). P*_guaB_* also contains a putative binding site for PurR that overlaps the core promoter region, and PurR downregulates expression of *guaB* (Meng *et al.*, 1990; Davies & Drabble, 1996). DnaA binds to a sequence overlapping the core promoter region and also downregulates transcription from P*_guaB_* (Tesfa-Selase & Drabble, 1996). It has also been shown that the rate of transcription from P*_guaB_* per unit cell mass increases as a function of increasing cellular growth rate (Davies & Drabble, 1996; Husnain & Thomas, 2008). This phenomenon is commonly referred to as growth rate-dependent control (GRDC) (Gourse *et al.*, 1996; Dennis *et al.*, 2004). GRDC of P*_guaB_* requires the UP element and sequences located upstream of the UP element (Husnain & Thomas, 2008).

**Fig. 1. f1:**
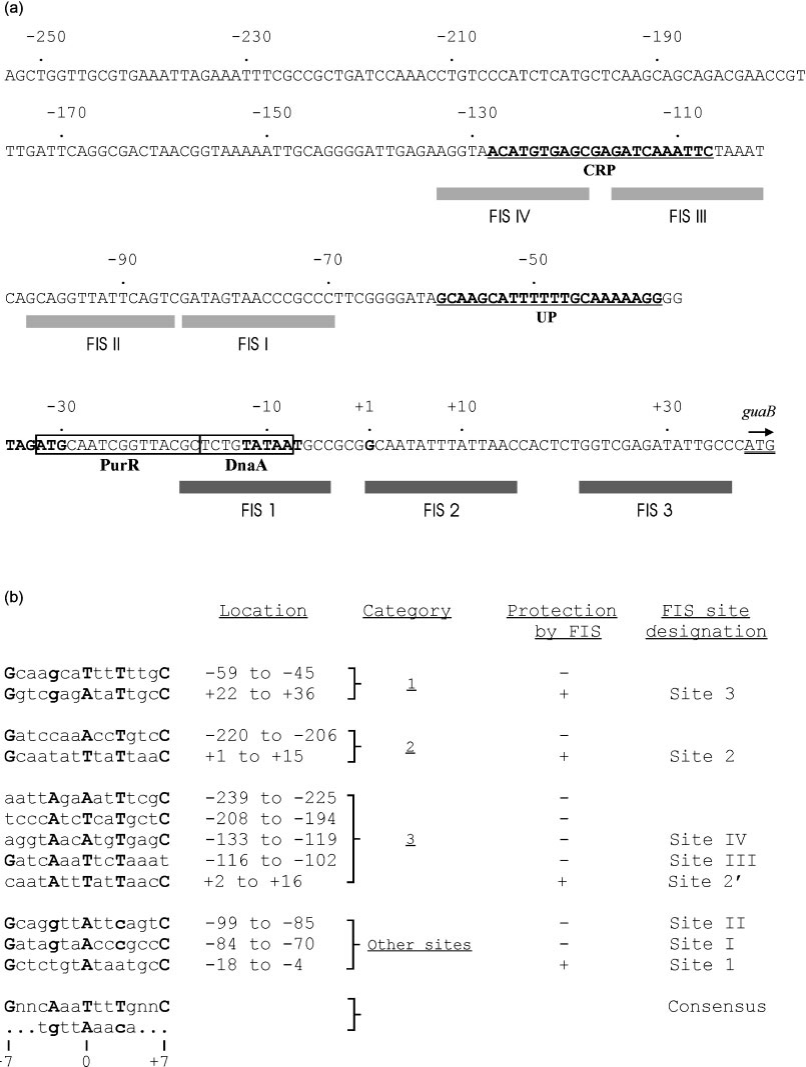
Identification of putative FIS sites at P*_guaB_*. (a) Sequence of the *guaB* promoter from positions −253 to +40 relative to the *guaB* transcription start site. Putative FIS sites I–IV, FIS sites 1–3, a putative CRP binding site centred at position −117.5 and binding sites for PurR and DnaA are indicated (Hutchings & Drabble, 2000). The core promoter elements (−35 and −10 regions), UP element, and the initiating nucleotide are also shown, in bold. Apart from FIS sites I–IV, putative FIS sites that have been shown not to bind FIS in this work are not indicated. For clarity, FIS site 2′ is not shown. (b) Candidate FIS binding sites were identified by comparing the consensus sequence for FIS (Ross *et al.*, 1999; Shultzaberger *et al.*, 2007) with P*_guaB_* sequences located from positions −253 to +36. The consensus sequence employed is indicated accordingly; alternative bases at each position of the consensus sequence are shown in the second line. Conserved bases at the five positions considered to be critical for FIS binding (positions −7, −3, 0, +3 and +7) are emboldened, and the most frequently occurring base is shown in upper case. ‘n' signifies any base. Candidate FIS sites are categorized according to their similarity to the consensus. Category 1 and 2 sites exhibit a 4/5 match at the critical positions, including positions +7 and −7, which are most strongly conserved among FIS sites. However, at category 2 sites, an infrequently occurring base is present at position −3, 0 or +3 (in the case of P*_guaB_*, such mismatches only occur at position −3). Category 3 sites also exhibit a 4/5 match at the critical positions but the mismatched base occurs at position +7 or −7. Other pertinent sites that do not fulfil the criteria for inclusion in categories 1–3 are shown as ‘other sites'. FIS sites that were protected by FIS in DNase I footprinting are as indicated.

The factor for inversion stimulation (FIS) regulates transcription by binding to highly degenerate 15 bp DNA sequences (Finkel & Johnson, 1992; Ross *et al.*, 1999). At some *E. coli* promoters, FIS activates transcription by contacting the C-terminal domain of the RNA polymerase (RNAP) *α* subunit (*α*CTD) (Aiyar *et al.*, 2002; McLeod *et al.*, 2002). FIS can also promote transcription by decreasing the negative superhelicity of DNA (Travers *et al.*, 2001). At other promoters, FIS downregulates promoter activity by binding to a site that overlaps or is located downstream from the RNAP binding site, or by forming a complex assembly with other nucleoid proteins (Gonzalez-Gil *et al.*, 1998; Browning *et al.*, 2000, 2004; Jackson *et al.*, 2004). A previous study identified four putative binding sites for FIS that are located upstream of the P*_guaB_* core promoter region (Hutchings & Drabble, 2000). The putative FIS binding sites are centred near positions −77, −92, −109 and −126 relative to the *guaB* transcription start, and were referred to as FIS sites I–IV, respectively (Fig. 1a[Fig f1]). FIS contributes to GRDC of the *thrU* and *pdxA* promoters, and is required for growth rate-dependent synthesis of 4.5S RNA, 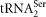 and 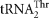 (Emilsson & Nilsson, 1995; Dong *et al.*, 1996). Moreover, transcription from the *fis* promoter is coupled to cellular growth rate (Mallik *et al.*, 2006). Cellular levels of FIS and FIS mRNA also change with the growth phase, and they increase dramatically upon entry into the mid-exponential growth phase (Appleman *et al.*, 1998; Ali Azam *et al.*, 1999; Mallik *et al.*, 2006).

In this work, we investigated whether FIS plays a role in the regulation of P*_guaB_*. We show that the putative FIS binding sites located upstream of the P*_guaB_* core elements do not recruit FIS (Hutchings & Drabble, 2000). Moreover, we demonstrate that FIS is recruited to three sites centred near −11, +8 and +29 relative to the *guaB* transcription start site, and all three sites are necessary for full FIS-mediated repression. We also show that FIS is not required for GRDC of P*_guaB_*.

## METHODS

### Strains and plasmids.

All strains were derivatives of the *E. coli* K-12 strain VH1000. Each strain contained a chromosomally integrated transcriptional fusion of *lacZ* to one of three P*_guaB_* derivatives: the full-length *guaB* promoter (i.e. strain VH1000G-253), extending from positions −253 to +36 relative to the *guaB* transcription start site [P*_guaB_* (−253 to +36)] (Husnain & Thomas, 2008), the P*_guaB_* (−253 to +10) promoter (i.e. strain VH1000G-25310, this work), which contains the same upstream end point as the full-length promoter but has a downstream end point at +10, and the P*_guaB_* (−69 to +36) promoter (i.e. strain VH1000G-69, this work), which has the same downstream end point as the full-length promoter but has an upstream end point at position −69 relative to the *guaB* transcription start site. Fusions were carried on *λ* prophage and were constructed using a system based on *λ*imm21 (Simons *et al.*, 1987; Rao *et al.*, 1994). Strain VH1000G-253Δ*fis* was made by introducing the *fis* : : *aadA* allele from strain JCB38841 (Ball *et al.*, 1992; Wu *et al.*, 1998) into VH1000G-253 by P1 transduction; strain VH1000G-69Δ*fis* was made by introducing the same allele into VH1000G-69. All plasmids contain P*_guaB_* derivatives that were inserted as *Eco*RI–*Hin*dIII fragments. Plasmids pBSG-253 and pBSG-133 are derivatives of pBluescript II KS containing the promoters P*_guaB_* (−253 to +36) and P*_guaB_* (−133 to +36), respectively. pUCG-253 is a derivative of pUC19 containing P*_guaB_* (−253 to +36). The plasmid pRLG770 has been described previously (Ross *et al.*, 1990). pRLG770 derivatives containing promoters P*_guaB_* (−253 to +36), P*_guaB_* (−133 to +36), P*_guaB_* (−59 to +36) and P*_guaB_* (−37 to +36) were constructed previously (Husnain & Thomas, 2008). pRLG770 derivatives pRLG-13321, pRLG-1331 and pRLG-25310 contain promoters P*_guaB_* (−133 to +21), P*_guaB_* (−133 to +1) and P*_guaB_* (−253 to +10), respectively.

### DNase I footprinting.

The *Eco*RI–*Xho*I DNA fragment in pBSG-253 was purified following electrophoresis in a 6 % acrylamide gel (Meng *et al.*, 2000), labelled at the downstream (*Xho*I) end with [*γ*-^32^P]ATP [>7000 Ci (2.59×10^14^ Bq) mmol^−1^, MP Biomedicals] and subsequently purified according to a published procedure (Husnain & Thomas, 2008). The *Eco*RI–*Xho*I fragment in pBSG-133 was labelled similarly at the upstream end. Labelled DNA fragment (4 nM) was incubated at room temperature for 30 min in a volume of 20 μl containing 20 mM HEPES (pH 8.0), 5 mM MgCl_2_, 50 mM potassium glutamate, 1 mM DTT, 20 μg ml^−1^ sonicated calf thymus DNA (GE Healthcare) and 5 % (v/v) glycerol, in the absence or presence of purified FIS protein. Purified FIS was a generous gift from T. Gaal and R. L. Gourse (University of Wisconsin–Madison). DNase I footprinting and DNA fragment separation were performed exactly as described previously (Husnain & Thomas, 2008). Footprints were visualized using a FujiFilm FLA3000 phosphorimager.

### Electromobility shift assay (EMSA).

A DNA fragment containing P*_guaB_* (−253 to +36) was amplified by PCR from pUCG-253, using primers pUC19(for) (5′-ACGTTGTAAAACGACGGCCAG-3′) and pUC19(rev) (5′-GCGCGGATCCATGACCATGATTACGCCAAGCT-3′). A DNA fragment containing the *rrnB* P1 promoter with FIS site I (positions −87 to +50 relative to the *rrnB* P1 transcription start site) and an *rrnB* P1 promoter derivative that did not contain a FIS site (positions −37 to +52 relative to the *rrnB* P1 transcription start site, and containing non-*rrnB* P1 sequences upstream to position −92) were PCR amplified from plasmids pRLG1616 and pRLG4720, respectively, using a forward primer with the sequence 5′-GTATCACGAGGCCCT-3′ and reverse primer RLG1620 (5′-GCGCTACGGCGTTTCACTTC-3′), both of which are vector-specific (Newlands *et al.*, 1991; Ross *et al.*, 1998; Meng *et al.*, 2001). PCR products were digested with *Hin*dIII, and purified following electrophoresis in a 6 % acrylamide gel (Meng *et al.*, 2000). Fragments were labelled at the *Hin*dIII end using [*α*-^32^P]dATP [3000 Ci (1.11×10^14^ Bq) mmol^−1^, MP Biomedicals] and DNA polymerase I Klenow fragment. Labelled DNA (final concentration 0.4 nM) was incubated at room temperature for 30 min in a volume of 10 μl containing 20 mM HEPES (pH 8.0), 5 mM MgCl_2_, 50 mM potassium glutamate, 1 mM DTT, 10 % (v/v) glycerol and 20 μg ml^−1^ sonicated calf thymus DNA (GE Healthcare), in the absence or presence of different concentrations of FIS. Samples were loaded (under tension) onto a 6 % acrylamide gel (37.5 : 1 acrylamide : bis acrylamide) containing 7.5 % (v/v) glycerol while running at ∼15 V cm^−1^, and gels were run for ∼1 h at 4 °C. Radiolabelled DNA was visualized using a FujiFilm FLA3000 phosphorimager.

### Measurement of transcription *in vitro*.

Multiple-round transcription reactions were performed as described previously, using supercoiled pRLG770 derivatives containing P*_guaB_* fragments (Husnain & Thomas, 2008). As a control, transcription was also measured from an *rrnB* P1 promoter derivative that did not contain any FIS sites (plasmid pRLG4238; Estrem *et al.*, 1998). FIS (250 nM) was incubated with DNA in reaction buffer at room temperature for 30 min. Transcription was initiated at 30 °C with 10 nM *E. coli* RNAP holoenzyme (Epicentre), and reactions were allowed to proceed for 20 min.

### Measurement of transcription *in vivo*.

Strains containing a chromosomally integrated P*_guaB_*-*lacZ* transcriptional fusion were employed in the measurement of promoter activity *in vivo*. Cells were inoculated from dense starter cultures into media that supported different cellular growth rates, as described previously (Husnain & Thomas, 2008). The *β*-galactosidase activity was determined following disruption of cells by sonication (Miller, 1972). To measure promoter activity at different stages of the growth cycle, cells were grown overnight in M9 minimal medium with 0.4 % (w/v) glucose, 0.8 % (w/v) Casamino acids and 5 μg thiamine ml^−1^, and inoculated into fresh medium to an OD_600_ of ∼0.01. Growth was monitored at OD_600_, and the *β*-galactosidase activity was measured at different points on the growth curve after cells were permeabilized with chloroform-SDS (Miller, 1972).

## RESULTS

### Identification of putative FIS sites at P*_guaB_*

The 15 bp consensus sequence for FIS [5′-Gnn(c/t)(A/g)(a/t)(a/t)(T/A)(t/a)(t/a)(T/c)(g/a)nnC-3′] contains five highly conserved positions (underlined) that are the most significant for the recruitment of FIS (Finkel & Johnson, 1992; Hengen *et al.*, 1997; Ross *et al.*, 1999; Shultzaberger *et al.*, 2007; Shao *et al.*, 2008). At each highly conserved position (hereafter referred to as a ‘critical’ position), an upper-case letter indicates the most strongly conserved base at that position, and a lower-case letter indicates a conserved base that is non-consensus. We employed the MatInspector program (Genomatix) to identify putative FIS sites at P*_guaB_* that matched the consensus in at least four out of the five critical positions (Quandt *et al.*, 1995). This was achieved by performing a sequence alignment of a modified consensus sequence (5′-GnnnRnnWnnYnnnC-3′, where R=A or G; W=A or T; Y=T or C; n=any base) with P*_guaB_* DNA sequences located between positions −253 and +36, and also with the reverse complement of this DNA sequence.

Using this approach, no sites were identified that contained the most highly conserved base at all five critical positions. However, we identified nine candidate FIS binding sites that contained the most highly conserved base at 4/5 critical positions (Fig. 1b[Fig f1]). All of these sites contained the consensus A or T base at the central position (position 0) and the conserved T residue at position +3. These sequences were subdivided into three categories. Category 1 sites contain bases that match the consensus at 4/5 critical positions, including the outer bases (positions −7 and +7) that are most strongly conserved among FIS sites and which are presumed to be bound by the D helices of FIS (Shultzaberger *et al.*, 2007). The remaining critical position (position −3) contained the alternative purine base G that occurred less frequently at that position. Two candidate FIS sites were identified that fell into this category (Fig. 1b[Fig f1]). Category 2 sites differ from category 1 sites in having a less frequently occurring C or T residue at position −3. Two additional sites fell into this category. Category 3 sites also contain bases that match the consensus at 4/5 critical positions. However, the mismatches occur at one of the highly conserved bases that are located at the outermost positions. Five sequences were classified as category 3 sites (Fig. 1b[Fig f1]). Two of them correspond to the previously identified putative FIS sites III and IV located upstream of P*_guaB_* (Hutchings & Drabble, 2000). Putative FIS sites I and II were not identified by this analysis as they harbour bases that match the consensus at only 3/5 of the critical positions, although they do include non-consensus but frequently occurring bases at the remaining two critical positions (positions −3 and +3) (Fig. 1b[Fig f1]).

### Analysis of FIS binding to P*_guaB_*

**Fig. 2. f2:**
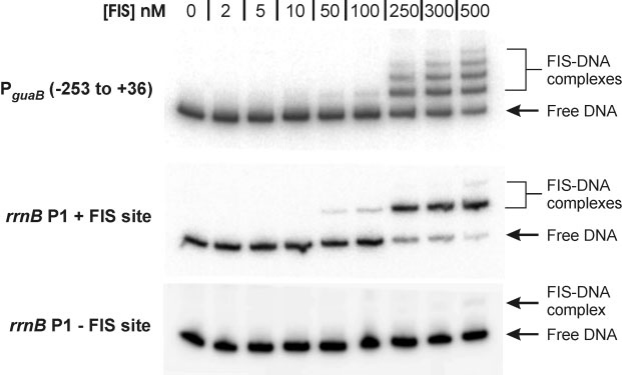
Analysis of FIS binding to P*_guaB_* by EMSA. EMSA was employed to compare the relative binding affinity of FIS for a DNA fragment containing P*_guaB_* (−253 to +36) with an *rrnB* P1 promoter derivative containing FIS site I (‘*rrnB* P1+FIS site’) and *rrnB* P1 containing no FIS sites (‘*rrnB* P1–FIS site’). The concentration of FIS in each binding reaction is indicated above the corresponding gel lane.

EMSA was employed to determine whether FIS can bind to a DNA fragment containing the *guaB* promoter. As a comparison, binding of FIS to the *rrnB* P1 promoter, which is known to bind FIS under physiological conditions, was also analysed. The *rrnB* P1 promoter fragment employed contained the promoter-proximal FIS site, i.e. FIS site I (Ross *et al.*, 1990; Bokal *et al.*, 1995). The minimum concentration of FIS required to observe FIS–DNA interactions at either P*_guaB_* (−253 to +36) or the *rrnB* P1 fragment by EMSA was 50 nM. Increasing the FIS concentration to 300 nM resulted in the formation of three different complexes between FIS and P*_guaB_* (Fig. 2). At this concentration of FIS, a larger fraction of the *rrnB* P1 promoter fragment was bound by FIS, but there remained only a single FIS–DNA complex, and no FIS–DNA complexes were observed at an *rrnB* P1 promoter derivative that lacked a FIS site (Fig. 2). At a FIS concentration of 500 nM, an additional FIS–DNA complex was observed at both P*_guaB_* and *rrnB* P1 harbouring FIS site I. As a complex was also observed with the promoter fragment that did not contain a FIS site, it is likely that the additional FIS–DNA interactions observed at 500 nM FIS are non-specific (Fig. 2). These results indicate that FIS binds to at least three sites at or near P*_guaB_* under similar conditions to those in which FIS specifically binds to *rrnB* P1, and thereby suggest that FIS is likely to bind to these sites under physiological conditions.

### Mapping the location of FIS binding sites at P*_guaB_* by DNase I footprinting

**Fig. 3. f3:**
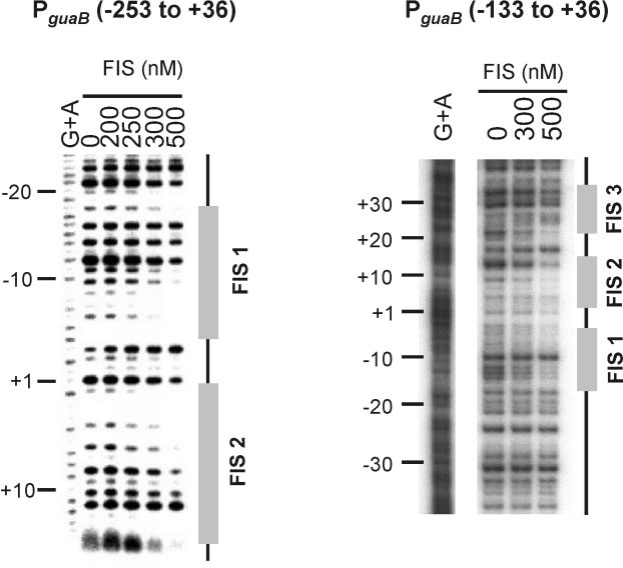
Mapping the location of FIS sites at P*_guaB_* by DNase I footprinting. A DNA fragment containing P*_guaB_* (−253 to +36) radiolabelled at the downstream end (relative to the *guaB* transcription start site), and a DNA fragment containing P*_guaB_* (−133 to +36) labelled at the upstream end, were employed in DNase I footprinting in the presence of different concentrations of FIS (as shown). Nucleotide positions are shown relative to the *guaB* transcription start site, and lanes containing the G+A ladder are indicated accordingly.

To determine the location of any FIS binding sites at P*_guaB_*, a DNA fragment extending from positions −253 to +36 of P*_guaB_* [i.e. P*_guaB_* (−253 to +36)] was radiolabelled at the downstream end, and DNase I footprinting was performed in the presence or absence of purified FIS. The results show that increasing the concentration of FIS up to 500 nM resulted in increased protection at two sites centred near positions −11 and +8 (FIS site 1 and FIS site 2, respectively) (Fig. 3). DNA fragments corresponding to P*_guaB_* sequences downstream of position +16 were not visible on this gel. To determine whether FIS bound to sequences downstream of position +16, a DNA fragment extending from positions −133 to +36 [i.e. P*_guaB_* (−133 to +36)] was radiolabelled at the upstream end for DNase I footprinting. The results show that, in addition to the protection observed at FIS sites 1 and 2, a third site (FIS site 3) centred near position +29 was also bound by FIS. FIS sites 2 and 3 were identified by comparison with the consensus as being more likely to recruit FIS (i.e. FIS site 3 is a category 1 site, and FIS site 2 is a category 2 site) (Fig. 1b[Fig f1]). However, protection of other category 1 and category 2 FIS sites that were identified by bioinformatic analysis was not observed. Interestingly, FIS site 1 contains mismatches to the consensus at two critical positions, and therefore was not identified by the bioinformatic analysis (Fig. 1b[Fig f1]). FIS did not bind to any of the previously predicted FIS sites (FIS sites I–IV). It should be noted that another FIS site (site 2′) is located overlapping site 2, with its centre shifted by one base pair downstream of the centre of site 2 (Fig. 1). As sites that contain both an A at position −4 and a T at position +4 are bound by FIS much less efficiently than are sites that lack a G at position −7 (or a C at +7) (Shao *et al.*, 2008), it is possible that site 2′ may be preferred by FIS over site 2.

### Role of FIS in the regulation of transcription from P*_guaB_ in vitro*

**Fig. 4. f4:**
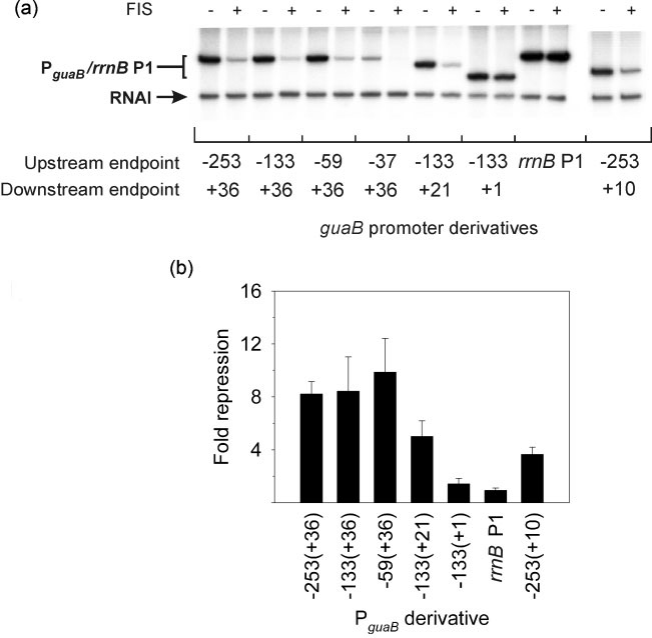
Role of FIS in regulation of transcription from P*_guaB_* *in vitro*. (a) Multiple-round *in vitro* transcription was employed to measure transcription from P*_guaB_* derivatives P*_guaB_* (-253 to +36), P*_guaB_* (-133 to +36), P*_guaB_* (-59 to +36), P*_guaB_* (-37 to +36), P*_guaB_* (-133 to +1), P*_guaB_* (-133 to +21) and P*_guaB_* (-253 to +10) in the presence (+) and absence (−) of 250 nM FIS. Promoter endpoints are as indicated. P*_guaB_* (-133 to +21) lacks FIS site 3, and P*_guaB_* (-133 to +1) lacks FIS sites 2 and 3. As a control, transcription was also measured from an *rrnB* P1 promoter derivative that did not contain any FIS sites. All promoters were cloned in pRLG770 and supercoiled DNA was used for the assays. The vector-encoded replication repressor, RNAI (∼110 nucleotides), is also indicated. (b) The repression afforded by FIS at each promoter is shown. The fold repression by FIS was calculated by dividing the activity in the absence of FIS by the activity in its presence. Values are the mean (with standard deviation) of three independent experiments. The activity of P*_guaB_* (-37 to +36) in the presence of FIS was too low to quantitate and hence a value for the fold repression was not obtained.

To determine the role of FIS in the regulation of P*_guaB_*, multiple-round transcription reactions were performed in the presence or absence of 250 nM FIS. Transcription was measured from P*_guaB_* (−253 to +36) and shorter derivatives [P*_guaB_* (−133 to +36), P*_guaB_* (−59 to +36), P*_guaB_* (−37 to +36), P*_guaB_* (−133 to +21), P*_guaB_* (−133 to +1) and P*_guaB_* (−253 to +10)] (end points as indicated). Addition of FIS to the transcription reaction resulted in ∼8–10-fold repression of transcription from P*_guaB_* (−253 to +36). Under the same conditions, there was no repressive effect of FIS on transcription from the *rrnB* P1 promoter (Fig. 4a, b[Fig f4]). This indicates that the repression of P*_guaB_* afforded by FIS at a concentration of 250 nM was due to a site-specific FIS–DNA interaction. Deletion of sequences upstream of the P*_guaB_* UP element [i.e. P*_guaB_* (−59 to +36)] did not lead to any significant change in the degree of repression afforded by FIS, confirming that putative FIS sites I–IV do not play a role in the regulation of P*_guaB_* by FIS. Deletion of the P*_guaB_* UP element [P*_guaB_* (−37 to +36)] gave rise to an undetectable level of transcripts from P*_guaB_* in the presence of FIS, which meant that the fold repression afforded by FIS could not be determined. Deletion of FIS site 3 [i.e. P*_guaB_* (−133 to +21)] led to decreased repression by FIS (approximately sixfold repression), and deletion of both FIS site 2 and FIS site 3 [i.e. P*_guaB_* (−133 to +1)] gave rise to an approximately twofold repression by FIS (Fig. 4b[Fig f4]). As P*_guaB_* (−133 to +1) retains FIS site 1 and is still subject to some degree of repression by FIS, these results indicate that FIS sites 1–3 each contribute to repression of P*_guaB_*, and that FIS site 1 can function independently of the other FIS sites. A P*_guaB_* derivative harbouring a deletion of FIS site 3 and deletion of the downstream six bases of FIS site 2 [i.e. P*_guaB_* (−253 to +10)] was subject to an approximately fourfold repression by FIS, indicating that FIS site 2 had not been completely inactivated (Fig. 4b[Fig f4]).

### FIS is not required for growth rate-dependent control of P*_guaB_*

**Fig. 5. f5:**
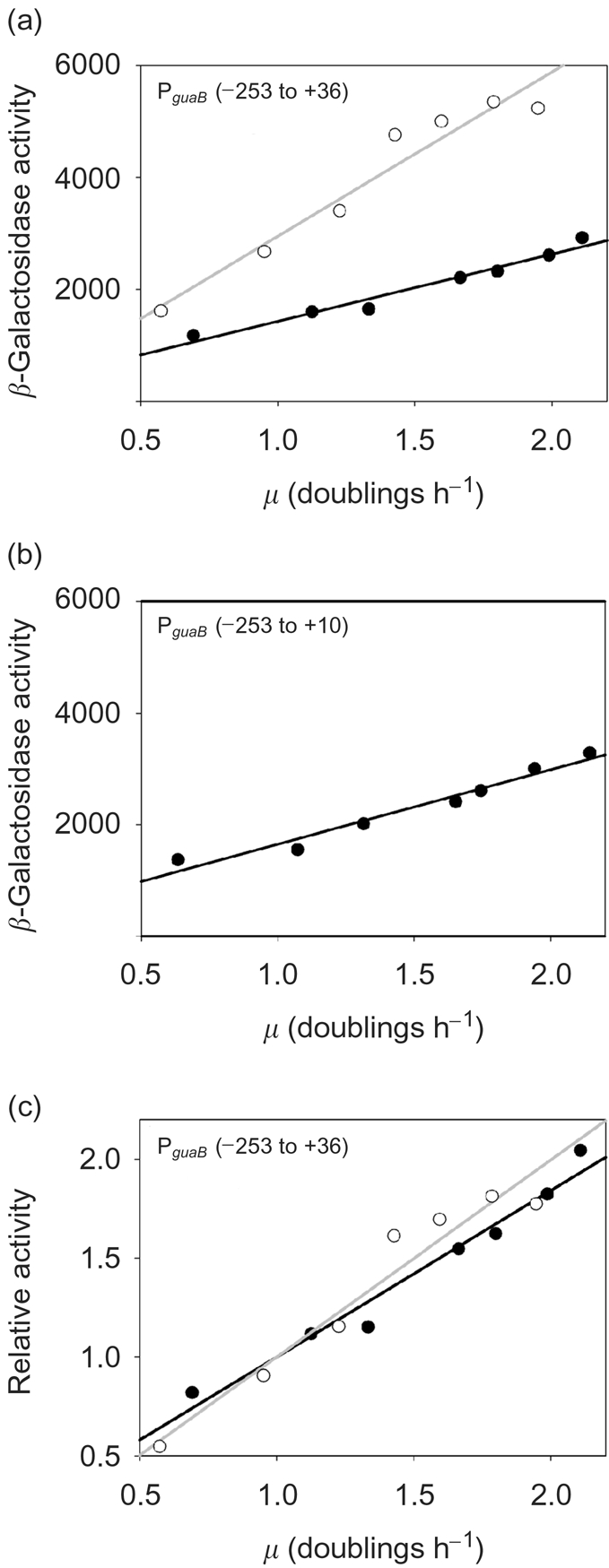
GRDC of P*_guaB_*. (a, c) GRDC of a wild-type strain (•) or a strain containing the *fis* : : *aadA* allele (○), harbouring a fusion of P*_guaB_* (−253 to +36) to *lacZ*, was analysed. (b) GRDC of a wild-type strain harbouring a P*_guaB_* (−253 to +10)-*lacZ* fusion was also measured. Strains were grown at different cellular growth rates to an OD_600_ of 0.34–0.45, whereupon the *β*-galactosidase activity was determined. The promoter activity for P*_guaB_* (−253 to +36) in the presence and absence of functional *fis* is given both in Miller units (*β*-galactosidase activity) and expressed as a ratio to the activity at 1 doubling per hour (relative activity). The magnitude of the gradient in plots of relative promoter activity versus doublings per hour is proportional to the degree of GRDC. Each data point represents the mean promoter activity or mean growth rate. The mean was calculated using data obtained from three independent experiments.

GRDC of P*_guaB_* (−253 to +36) was measured in a wild-type strain background and in a strain that harboured a deletion in the *fis* gene. To determine whether FIS site 3 is important for GRDC of P*_guaB_*, activity of P*_guaB_* (−253 to +10) was also analysed. In exponentially growing wild-type *E. coli* cells, the activity of P*_guaB_* (−253 to +36) increased approximately twofold with every doubling of the growth rate as, shown previously (Fig. 5a, c[Fig f5]; Davies & Drabble, 1996; Husnain & Thomas, 2008). In an otherwise isogenic *fis* strain, the activity of P*_guaB_* (−253 to +36) was higher than in the wild-type at all growth rates and was more pronounced at faster growth rates (i.e. an approximately twofold increase in activity was observed at the fastest growth rate) (Fig. 5a[Fig f5]). However, the degree of GRDC was similar to that observed in a wild-type strain (i.e. in both cases, a doubling of the growth rate corresponded to an approximately twofold increase in promoter activity) (compare plots of relative activity versus growth rate, Fig. 5c[Fig f5]). These results demonstrate that FIS is not required for GRDC of P*_guaB_*. Although FIS appears to downregulate transcription from P*_guaB_ in vivo*, deletion of FIS site 3 and part of FIS site 2 did not lead to a significant change in P*_guaB_* activity in exponentially growing wild-type cells in comparison to P*_guaB_* containing the full complement of functional FIS sites [i.e. a 1.87-fold increase in P*_guaB_* (−253 to +10) activity occurred for every doubling of the growth rate in comparison to a 1.84-fold increase for P*_guaB_* (−253 to +36) (Fig. 5a, b[Fig f5])]. These results suggest either that FIS site 1 is able to effect full repression *in vivo* (contrasting with the results obtained *in vitro*) or that FIS sites 1–3 may not contribute to repression of P*_guaB_ in vivo* under the conditions employed, and therefore the observed FIS-dependent repression is indirect.

### FIS is not required for growth phase-dependent regulation of P*_guaB_*

Previous studies indicate that FIS levels are elevated during the mid-exponential growth phase, and they decrease sharply as cells enter stationary phase (Appleman *et al.*, 1998). This phenomenon is responsible for the known contribution of FIS to growth-phase-dependent regulation of some promoters (Nilsson *et al.*, 1992; Appleman *et al.*, 1998; Mallik *et al.*, 2006; Bradley *et al.*, 2007). To test whether FIS-dependent regulation of P*_guaB_* varies with the growth phase, transcription from a P*_guaB_* derivative with an upstream end point of −69 that contained all three experimentally determined FIS sites [i.e. P*_guaB_* (−69 to +36)] was measured at different stages of growth in a wild-type strain, and in a strain that harboured a deletion in the *fis* gene. This promoter derivative was chosen as it lacks the putative CRP site centred near position −117.5 (Hutchings & Drabble, 2000). It has previously been shown that FIS represses the *crp1* promoter and this may alter cellular levels of CRP (Gonzalez-Gil *et al.*, 1998).

**Fig. 6. f6:**
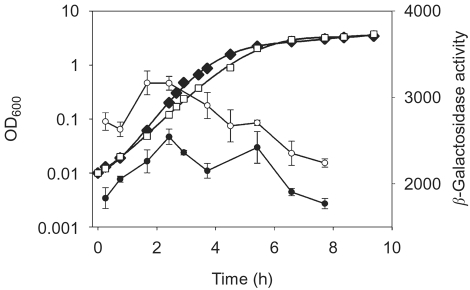
Growth-phase-dependent regulation of P*_guaB_*. A wild-type strain or a strain containing the *fis* : : *aadA* allele (Δ*fis*), each harbouring a fusion of P*_guaB_* (−69 to +36) to *lacZ*, were inoculated from a dense culture into fresh growth medium [M9 minimal medium containing 0.4 % (w/v) glucose, 0.8 % (w/v) Casamino acids and 5 μg thiamine ml^−1^] to an OD_600_ of ∼0.01. Samples were taken during different stages of growth and the *β*-galactosidase activity was determined. The promoter activity of the wild-type strain (•) and Δ*fis* strain (○) is given in Miller units (*β*-galactosidase activity). Values presented are the mean±sd, for three independent experiments. Cell density measurements (OD_600_) for single cultures that are representative of the growth curve for the wild-type strain (⧫) and the Δ*fis* strain (□), were plotted on a logarithmic axis.

In accordance with the results of the GRDC experiment, the activity of P*_guaB_* was higher in the *fis* background than in the wild-type strain throughout the course of the growth cycle. In the wild-type strain, P*_guaB_* activity increased by nearly 40 % as cells entered the mid-exponential growth phase (i.e. the promoter activity at an OD_600_ of ∼0.15–0.20 was 40 % higher than the activity at an OD_600_ of ∼0.012). The increase in activity in a *fis* strain over the corresponding part of the growth curve was less marked (i.e. there was a ∼16 % increase in promoter activity). Upon entry into stationary phase, there was a gradual decrease in the promoter activity in both strain backgrounds (Fig. 6). The results suggest that P*_guaB_* is subject to a degree of growth-phase-dependent regulation. However, there was no significant change in the transcription activity profile during the growth cycle when comparing the two strain backgrounds. Furthermore, P*_guaB_* activity peaks at the time that FIS levels are expected to be at their highest, and then falls off upon entry into stationary phase when FIS levels fall (Appleman *et al.,* 1998; Ali Azam *et al.*, 1999). These observations suggest that FIS does not significantly influence growth phase-dependent regulation of P*_guaB_* under the conditions employed.

## DISCUSSION

A previous study identified four adjacent putative FIS binding sites (FIS sites I–IV) located upstream of the UP element at P*_guaB_*. These sites were identified by comparison of the upstream P*_guaB_* sequence to a published consensus sequence for FIS (Finkel & Johnson, 1992, Hutchings & Drabble, 2000). By employing sequence alignment to compare P*_guaB_* sequences to a more representative consensus for FIS, we have identified four candidate FIS sites that bear a closer resemblance to this consensus than FIS sites I–IV (Ross *et al.*, 1999). Two of these sites, sites 2 (or 2′) and 3 (centred near positions +8 and +29 relative to the *guaB* transcription start site, respectively), are protected by FIS in DNase I footprinting experiments. One of the other two candidate FIS sites overlaps the UP element, and the remaining site is located at a distance upstream of the core promoter elements (centred at −213). Neither of these sites recruits FIS as judged by DNase I footprinting. Putative FIS sites I–IV also do not bind FIS. Interestingly, we show that a site centred at position −11, which was not identified as a likely FIS site, also recruits FIS. This suggests that although bioinformatic analyses are useful when searching for sites that are bound by FIS, they may not be useful in identifying some FIS binding sites that exhibit a weak match to the consensus.

At the *tyrT* promoter, FIS binding to sites II and III (centred at positions −91 and −122, respectively) cooperatively affects the binding of FIS to site I centred at −71 bp upstream of the transcription start site (Lazarus & Travers, 1993; Pemberton *et al.*, 2002). As the B-form of DNA has a periodicity of 10.6 bp, this places these three sites on the same face of the DNA helix, with the centres of sites I and II separated by 21 bp. This suggests that cooperative interactions require adjacent FIS dimers to be positioned on the same face of the DNA, two turns of the DNA helix apart. At the *rrnB* P1 promoter, FIS sites I–III are centred at positions −71, −102 and −143, respectively, also placing them approximately on the same face of the DNA helix. However, FIS does not bind to *rrnB* P1 cooperatively in the absence of RNAP, and it is noteworthy that the central positions of these sites are separated by 32 or 42 bp (i.e. not 21 bp). Interestingly, at P*_guaB_*, FIS sites 2 and 3 are positioned 22 bp apart (centre to centre) which will also place them on the same face of the DNA helix and a similar distance apart as FIS sites I and II at the *tyrT* promoter. However, the centres of FIS sites 1 and 2 at P*_guaB_* are 18 bp apart, making it unlikely that they are located on the same face of the DNA. Therefore, it is possible that FIS binds cooperatively to sites 2 and 3, but it appears less likely that occupancy of sites 2 and/or 3 stimulates binding of FIS to site 1.

Consistent with the location of functional FIS sites, we show that FIS represses transcription from P*_guaB_* ∼8–10-fold *in vitro*. Deletion of FIS site 3 results in partial relief of repression, and deletion of FIS sites 2 and 3 together further relieves repression *in vitro*. The residual FIS-mediated repression of the *guaB* promoter fragment containing the +1 downstream end point is likely to occur through interactions with site 1, and would suggest that binding of to site 1 does not require cooperative interactions with FIS dimers bound to adjacent sites. The binding of FIS to site 1 is likely to sterically hinder the recruitment of RNAP to P*_guaB_*, as observed at the *crp1* promoter (Gonzalez-Gil *et al.*, 1998). The role of FIS sites 2 and 3, which together exert the most influence on transcription from P*_guaB_ in vitro*, is less clear, although it is likely that the role of FIS site 3 is to stimulate binding of FIS to site 2, which in turn may play more of a direct role in repression. Our results suggest that the presence of FIS should decrease RNAP binding to P*_guaB_*. However, DNase I footprinting experiments carried out in the presence of both FIS and RNAP were inconclusive (data not shown).

Although our results demonstrate that FIS represses transcription from P*_guaB_ in vitro*, evidence for direct repression by FIS *in vivo* was not obtained (i.e. deletion of FIS sites 2 and 3 did not result in increased P*_guaB_* activity in wild-type exponentially growing cells). This is consistent with the results of a chromatin immunoprecipitation (ChIP)-chip analysis carried out under similar conditions, in which FIS binding at P*_guaB_* was not detected (see supplementary data in Grainger *et al.*, 2006). However, in a *fis* strain we observed an increase in the activity of the *guaB* promoter in derivatives containing all three FIS sites in the presence (P*_guaB_* (−253 to +36)) or absence [P*_guaB_* (−69 to +36)] of the putative CRP site centred at −117.5. This rules out the possibility that the effect of deleting *fis* on *guaB* promoter activity is mediated by changes in CRP abundance [FIS has been shown to modulate transcription of *crp* (Gonzalez-Gil *et al.*, 1998)]. However, it is possible that the change in transcription activity of P*_guaB_* in a *fis* background occurs as a result of altered regulation of P*_guaB_* by a transcription factor other than CRP, for example H-NS or HU (Claret & Rouviere-Yaniv, 1996; Falconi *et al.*, 1996) or by changes in supercoiling (Schneider *et al.*, 1997; Weinstein-Fischer *et al.*, 2000). Another possible explanation is that the potential relief of P*_guaB_* repression that occurs upon deleting FIS sites 2 and 3 is masked *in vivo* through an alternative compensatory regulatory mechanism. A less likely explanation, in view of the poor match to the consensus FIS binding site, is that FIS binding to site 1 mediates full FIS-mediated repression *in vivo*.

A previous study has shown that the P*_guaB_* UP element, and sequences located further upstream, are required for GRDC of P*_guaB_* (Husnain & Thomas, 2008). Our results show that FIS does not play a role in GRDC at this promoter, thereby implying that a different cellular factor is required for conferring GRDC on P*_guaB_* (Emilsson & Nilsson, 1995; Dennis *et al.*, 2004; Paul *et al.*, 2004). Experiments are under way to uncover the identity of this factor(s). Our results also suggest that P*_guaB_* is subject to growth phase-dependent control. However, although levels of FIS protein are also subject to growth phase-dependent control, it does not appear to play an important role in growth phase-dependent control at P*_guaB_*. Thus, the physiological role of FIS at the *guaB* promoter remains to be elucidated.

## Abbreviations

- CRP, cAMP receptor protein
- EMSA, electromobility shift assay
- FIS, factor for inversion stimulation
- GRDC, growth rate-dependent control
- RNAP, RNA polymerase
